# Cod-Liver Oil and Chemistry

**Published:** 1895-09

**Authors:** 


*Cod-Liver Oil and Chemistry.* By F. PECKEL MÖLLER, Ph.D. Pp. cxxiii., 508. London: PETER MÖLLER. 1895.

It is equally difficult to understand the reason for associating these two subjects, whether the interesting account of the Norwegian fisheries be regarded as a preface to the systematic text-book of organic chemistry, or the latter a voluminous appendix to the engrossing description of the Maelström, the contour of the Atlantic bottom, and the ethnological peculiarities of the hardy Norseman. It is perhaps safe to assume that the whole work is the outcome of a legitimate desire to improve the acquaintance of medical men and others with cod-liver oil in general, and with that elaborated by Messrs. Möller in particular. Regarded separately, however, the two sections of this book are full of interest, and by no means devoid of originality.

The first part, treating of the origin, nature and uses of the oil, contains much valuable information. After a general survey of the localities in which the fish are found, and the method of catching them and rendering the oil, there follows a copious synopsis of scientific research on the composition of the latter from the earliest days to the present time. The practical outcome of the latest work is the recognition of the necessity for rendering the oil without exposure to air; and the experimental evidence upon which this newest process of manufacture and the author's explanation of the special nutritive value of this food are based is interesting alike to the prescriber and to the pure chemist.

The characteristic feature of the second part is the wealth of diagrammatic illustration: and although there is a constant danger of taking the representations to mean more than is intended by the author, there can be no doubt whatever that some such contrivance is necessary to convey to the average mind some conception of the frequently complex structure of the organic molecule. The accompanying text is couched in language remarkably clear and intelligible, if now and then a little playful, and the author is careful to point out where, as in tracing the connection between the physical characters of compounds and their molecular structure, he is inclined to differ from orthodox views. On the whole it is doubtful whether the student could find a more instructive guide to the mysteries of stereochemistry.

